# Optimization and Sensitivity Analysis of the Cutting Conditions in Rough, Semi-Finish and Finish Honing

**DOI:** 10.3390/ma15010075

**Published:** 2021-12-23

**Authors:** Irene Buj-Corral, Lourdes Rodero-de-Lamo, Lluís Marco-Almagro

**Affiliations:** 1 Department of Mechanical Engineering, Barcelona School of Industrial Engineering (ETSEIB), Universitat Politècnica de Catalunya (UPC), 08028 Barcelona, Spain; 2 Department of Statistics and Operations Research, Barcelona School of Industrial Engineering (ETSEIB), Universitat Politècnica de Catalunya (UPC), 08028 Barcelona, Spain; lourdes.rodero@upc.edu (L.R.-d.-L.); lluis.marco@upc.edu (L.M.-A.)

**Keywords:** sensitivity analysis, honing, roughness, tool wear, material removal rate, optimization, desirability function, mixture design

## Abstract

Honing processes are currently employed to obtain a cross-hatched pattern on the internal surfaces of cylinders that favors oil flow in combustion engines or hydraulic cylinders. The main aim of the present paper is to optimize the machining conditions in honing processes with respect to surface roughness, material removal rate and tool wear by means of the desirability function. Five process variables are considered: grain size, density, pressure, linear speed and tangential speed. Later, a sensitivity analysis is performed to determine the effect of the variation of the importance given to each response on the results of the optimization process. In the rough and semi-finish honing steps, variations of less than 5% of the importance value do not cause substantial changes in the optimization process. On the contrary, in the finish honing step, small changes in the importance values lead to modifications in the optimization process, mainly regarding pressure. Thus, the finish honing phase is more sensitive to changes in the optimization process than the rough and the semi-finish honing phases. The present paper will help users of honing machines to select proper values for the process variables.

## 1. Introduction

Honing is an abrasive machining process in which a honing head provided with abrasive stones combines alternate linear movement with rotation in order to machine the internal surfaces of cylinders. The main goal of honing is to obtain a cross-hatched pattern with channels that favor oil flow in combustion engines or hydraulic cylinders. Several authors have indicated the key role of the surface topography of the cylinders’ liners on the friction coefficient in the piston/cylinder assembly as well as on the amount of oil consumption. Thus, selecting proper honing parameters can reduce the emission of toxic compounds during the operation of combustion engines [1,2,3].

Some researchers have studied the honing process by means of statistical models. For example, Troglio [4] considered the grain size of the abrasive, lubricating oil and workpiece material as variables, and studied different roughness parameters, such as average roughness Ra and parameters of the Abbott–Firestone curve (Rk, Rpk, Rvk, Mr1, Mr2). Kanthababu et al. [5] varied rotation speed, linear speed, pressure, honing time and plateau-honing time. Responses were roughness parameters of the Abbott–Firestone curve. Roughness was mainly influenced by pressure and honing or plateau-honing time. Wos and Michalsky [6] found that main roughness parameters improving aircraft piston engine performances (output power, torque, fuel consumption and total efficiency) are Rvq and the linear triangle area for valleys A2, although they provide a higher oil consumption and greater emissions. More recently, Vrac et al. [7] obtained exponential models for roughness and material removal rate as a function of process parameters, such as pressure or speed. In another study with diamond stones of grain size 151 and 181, they found that pressure was the most influential factor on roughness, followed by cutting speed and feed [8]. Vrabel et al. [9] analysed the influence of cutting speed, machining allowance and stone pressure on surface roughness, specifically on roughness parameters, the height of peaks (CR), the depth of the profile (CF) and the relative height of the holes (CL). On the other hand, Buj-Corral et al. searched for statistical models for both roughness and material removal rate in rough honing as a function of the main process parameters. They found that, in the range studied, abrasive grain size and pressure were the main parameters influencing both roughness and material removal rate [10]. Material removal rate values between 0.015 and 0.020 mm/s (0.090 and 0.120 cm/min) were found by Szabo [11] using cubic boron nitride (cBN) stones. In rough honing, Bai et al. [12] observed that the material removal rate increases with circumferential speed, reciprocate speed and cross-hatch angle, but decreases when the two speeds take too high values.

The semi-finish process was also studied, in which, as a general trend, roughness and material removal rate increase with grain size and density [13]. The finish honing process has been less well studied in the literature. For instance, Arantes et al. [14] compared the surface finish obtained in both conventional honing and flexible honing processes, including amplitude parameters, Rk family parameters, volume and feature parameters. In finish honing, Bai et al. [12] found that surface finish in finish processes improves when circumferential speed increases. Cabanettes et al. [15] studied the relationship between tool wear and different roughness parameters. They reported that only areal reduced summit height, arithmetic mean summit curvature and core roughness are correlated with tool wear.

Multi-objective optimization by means of the desirability function was developed by Derringer and Suich [16]. It has been applied in the past to machining processes, such as turning [17,18] or milling [19,20]. As for abrasive machining processes, Mukherjee et al. employed the desirability function and a metaheuristic technique for optimal process design [21]. Regarding honing processes, Lawrence and Rammamoorthy used robust process design and gray-relational analysis to define recommended values for process parameters in order to obtain required values for roughness Rz, roughness parameters from the Abbott–Firestone curve and honing angle, for three honing stages: rough, semi-finish and plateau-honing [22]. Nguyen et al. [23] carried out multi-response optimization of finishing honing with respect to surface finish and production rate. They noticed that both surface roughness and machining time depended mainly on grit size, followed by tangential speed and linear speed.

Sensitivity analysis is usually carried out in optimization problems, in order to assess the effect of the modification of either the objective function or the variables on the optimized values [24]. Different methods have been used in the past for performing sensitivity analysis after optimization. For example, Arsezer defined a methodology that consists of varying the parameters of the desirability function and analyzing their effect on desirability [25]. Malenovic et al. used a similar methodology for performing sensitivity analysis on the results of multi-objective optimization in a microemulsion LC system, and found the most sensitive parameters among importance, weight and ranges of the different responses [26]. In turning processes, Souza Rocha et al. [27] optimized tool life, surface roughness Ra and the ratio material removal rate/cutting force as a function of cutting speed, feed rate and depth of cut. They found that the weights used in the multi-objective optimization process influence the prediction variance. Mudhukrishnan et al. [28] optimized drilling parameters, such as spindle speed, feed and drill material, with respect to thrust force and torque, and performed a sensitivity analysis to assess the impact of control variables on the responses.

On the other hand, mixture design is a methodology that allows different kinds of mixtures to be tested; for example, in the formulation of cement or concrete pastes [29] or in the food industry [30]. This method is usually employed to determine the best composition of a certain product. In the manufacturing area, for example, Misra et al. [31] employed mixture design to find the optimal electrolyte composition in electrochemical honing of gears.

In the present paper, regression models were obtained for average roughness Ra, material removal rate and tool wear for the three steps of the honing process, namely, rough, semi-finish and finish honing. Afterwards, multi-objective optimization was carried out by means of the desirability function. Importance values were defined for each response in the three honing steps, according to users’ requirements. However, the selection of certain importance values for the responses could affect the results of the optimization process. Thus, in order to test the influence of the importance values on the optimal values of the variables in this work a sensitivity analysis was carried out. Mixture design was used to define different importance values to be tested. In order to assess the variability, the coefficient of variation CV was calculated for each response, considering different percentages of variation of the importance values.

This paper has two essential contributions. First, the recommendations for selecting the most appropriate parameters in each honing operation (rough, semi-finish and finish). Second, the final guidelines on how to define the importance of each parameter for the multi-objective optimization in each of the phases of the honing process.

## 2. Materials and Methods

### 2.1. Honing Experiments

**Figure 1:**
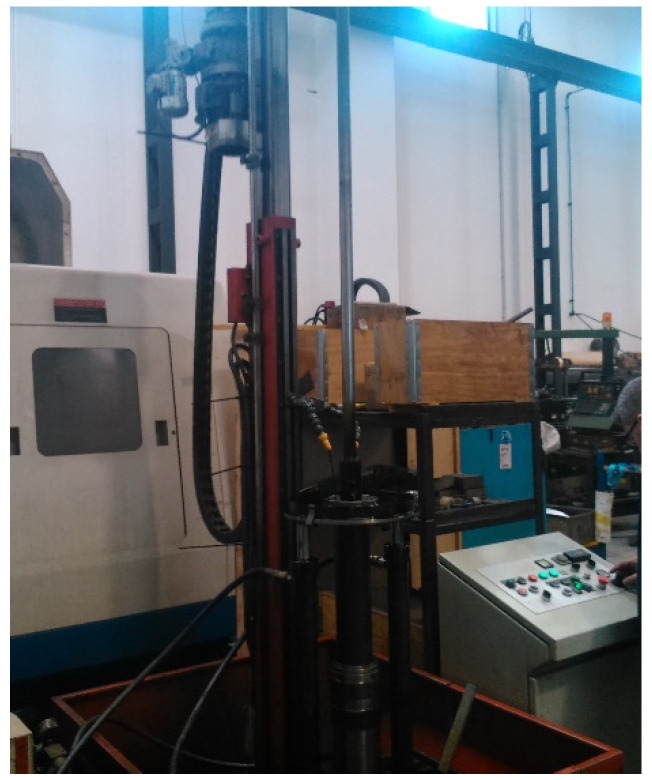
Industrial honing machine used in the experiment.

Steel St-52 cylinders of 80 mm interior diameter and 390 mm length were machined in a Honingtec industrial machine (Honingtec S.A., Els Hostalets de Balenyà, Spain). This material is usually employed to manufacture hydraulic cylinders. Figure 1 shows the industrial machine used. 

A central composite design was used to define the experiments in each one of the honing steps (rough, semi-finish and finish), which is explained in Section 2.5. Honing time was 30 min in all experiments. Two replicates were performed for each experiment.

**Figure 2:**
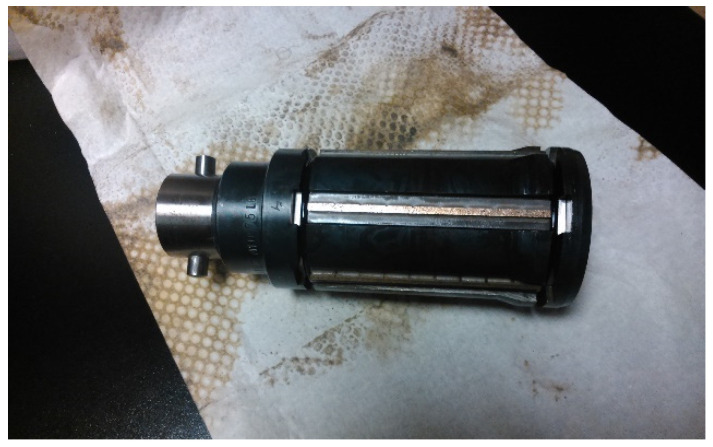
Honing head used in the experiment.

Cubic boron nitride (cBN) honing stones were used with metallic bonds. Figure 2 depicts the honing head employed.

For each of the three experimental designs, three responses were measured: roughness (Ra), material removal rate (Qm) and tool wear (Qp).

### 2.2. Roughness Measurement

**Figure 3:**
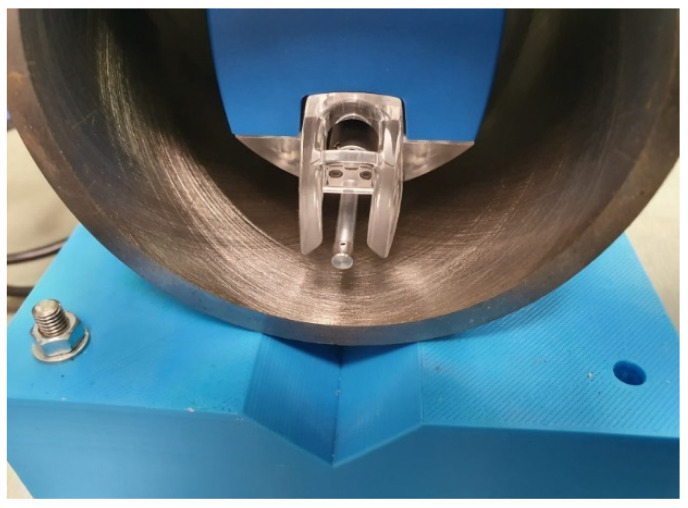
Contact roughness meter.

Arithmetical mean roughness Ra was measured with a Hommel-Etamic W5 contact roughness meter (Hommel-Etamic GmbH, Villingen-Schwenning, Germany), according to standard ISO 4287 [32] (Figure 3). 

Nine measurements were taken along a diametral circumference in the internal surface of cylinders at a distance of 195 mm from the end of the cylinders. The average value of the nine measurements was calculated. The cut-off length was 0.8 mm and the measuring length was 4 mm. 

### 2.3. Material Removal Rate Measurement

The material removal rate Qm was measured by means of the weight difference of the workpieces, before and after the honing test. Workpieces were weighed with a Kern FCB 3K0.1 scale (Kern &Sohn GmbH, Balingen, Germany).

Qm is defined as the volume of material removed in cm^3^ per min and per unit area of abrasive wheel in cm^2^. Qm in cm/min is calculated as follows (Equation (1)):

(1) $$\mathrm{Qm}=\frac{V}{S\cdot t}$$

where V is removed volume in cm^3^, S is abrasive surface in cm^2^ and t is honing time in min.

The removed volume V in cm^3^ is calculated from the weight W of the workpiece before and after honing (Equation (2)).

(2) $$V=\frac{\mathrm{Wi}-\mathrm{Wf}}{\rho }$$

where Wi is initial weight of the cylinder in g, Wf is the final weight of the cylinder in g, and ρ is the density of the cylinder in g/cm^3^.

### 2.4. Tool Wear Measurement

Tool wear Qp in cm^3^/min is calculated as follows (Equation (3)):

(3) $$\mathrm{Qp}=\frac{\mathrm{Vp}}{t}$$

where Vp is the volume of stone removed during honing (cm^3^), obtained with the initial and final weight of the stone and with the density of the stone, and t is the honing time in min.

### 2.5. Design of Experiments (DOE)

For each honing step, a central composite design was conducted in order to be able to obtain second order models for the responses. Minitab statistical software version 19, (Minitab LLC, State College, PA, USA) was used. The cube experimental runs were defined as a fractional factorial design 2^5−1^ with 16 runs. The axial runs were defined with 10 face-centered points plus three central points. Table 1 shows the variables and levels employed for rough, semi-finish and finish operations.

As can be seen in Table 1, the same levels were used for pressure, tangential speed and linear speed for the three honing steps. The values for the different parameters were selected according to the manufacturers’ recommendation and to the literature. For instance, Vrac et al. [7] recommended grain size 181 and 151 in normal honing. These values lie within the range that was selected in the present work for rough honing. Grain size and abrasive density usually decrease as the honing process advances in order to achieve finer and finer surfaces.

### 2.6. Multiobjective Optimization

In the present paper, the desirability function method was used to carry out multiobjective optimization [14]. 

The process searches for a combination of the factors that gives the best possible compromise for all the factors. This is achieved following these steps:

1. The individual desirability function for each response (di) is obtained.
2. The composite desirability function (D) is computed combining all the individual desirability functions, di, and considering the importance of each individual response.
3. The values of the factors that maximize the composite desirability function (D) are finally found.

The individual desirability functions map each one of the responses onto a value ranging from 0 to 1 (0 meaning that the level of the response is not what was wanted; 1 meaning that the level of the response is most preferred, the target). The formula depends on whether one wants to minimize the response, maximize the response or set the response to a target. In our study, we want to minimize roughness, Ra, and tool wear, Qp, and maximize the material removal rate, Qm. 

**Figure 4:**
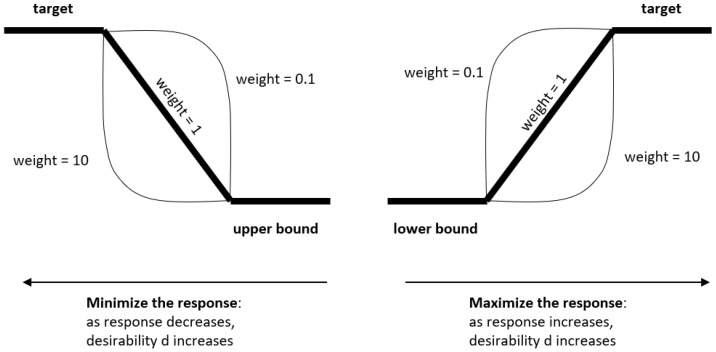
Desirability functions when minimizing (left) and maximizing (right) the response.

Figure 4 shows the shape of the function when minimizing (left) or maximizing (right) the response. In our study, we use the target and upper and lower bounds as the maximum and minimum response values obtained, depending on the situation. A weight of 1 was used in all cases, corresponding to the use of a linear function.

The composite desirability function D is computed using the formula shown in Equation (4).

(4) D=∏diImpi1IMP

Imp_i_ is the importance given to response i. IMP is the sum of all importance values, $\sum {\mathrm{Imp}}_{i}$. One can set the importance for each response so that the sum is one, thus simplifying the formula and giving the idea that the importance for each response is a percentage of importance. 

The importance values for each of the three responses in this study are shown in Table 2. They were selected from previous honing experiments. The following criteria were employed: in rough operations it is important to remove as much material as possible, while in finish operations surface finish is crucial. Thus, the importance values increase for roughness in subsequent honing operations, while they decrease for material removal rate and tool wear. In other words, in rough honing high importance values of Qm and Qp, as well as low values for Ra, are recommended. On the contrary, in finish honing high importance values are required for Ra and low values for Qm and Qp.

One of the main objectives of this study is assessing to what extend the results are dependent on the importance given to each of the responses. To achieve this objective, the importance of each response was later varied, in order to perform a sensitivity analysis of the optimization process (Section 2.7).

### 2.7. Sensitivity Analysis

The purpose of the sensitivity analysis is to determine the effect of a certain change in the importance values of the responses on the optimal values of the variables that are obtained from the multi-objective optimization. In order to achieve this, the values of the importance for the different responses were varied from the initially defined values in Table 2 with the help of a mixture design. Values of importance were varied from a slight degree (1%) to a considerable degree (15%) (the higher the variation in the initial importance values, the higher the expected impact on the optimization results). 

Mixture designs are special experiments in which the product being studied is composed of different ingredients. These ingredients cannot be modified independently: if the percentage of one ingredient in the formula increases, the percentages of others must decrease, as the total always sums to 1 [29]. These experiments are commonly used in pharma or food investigations. We have used a mixture design to change in an organized and balanced way the importance of each response in our optimization problem. 

**Figure 5:**
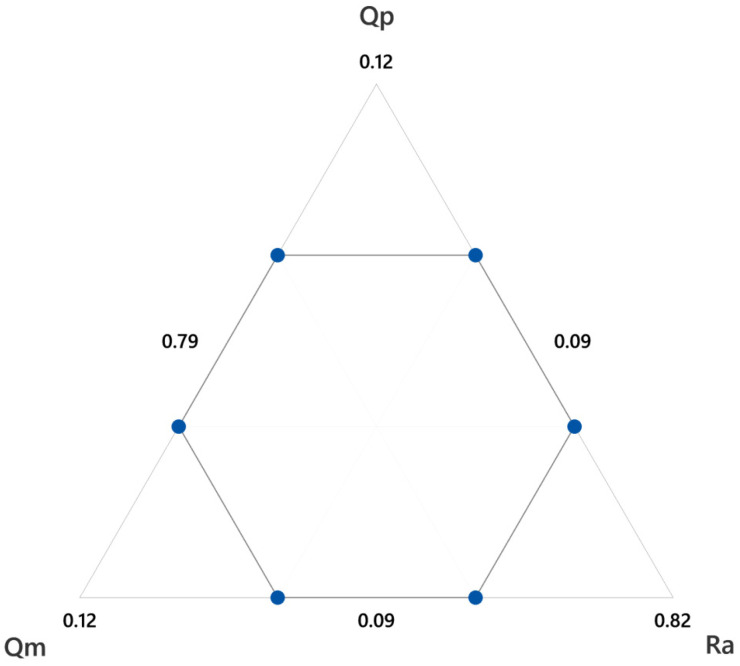
Optimization runs performed for the sensibility analysis in the finish step, with a 1% variation of the importance.

For instance, Figure 5 shows the experiments performed for the finish step. The central point corresponds to the initial importance values shown in Table 2 (Ra = 0.8, Qm = 0.1, Qp = 0.1). The other points are slight variations of these importance values, always summing to 1.

For each one of the runs coming from the mixture design we have a combination of values of the variables Gs, De, Pr, Vt and Vl that globally optimize the three responses. In order to see the extent to which these values vary depending on the run, the coefficient of variation CV was calculated for each variable, Gs, De, Pr, Vt and Vl, and for each percentage of variation of importance (1%, 3%, 5%, 10%, 15%).

## 3. Results and Discussion

### 3.1. Regression Models

For each response and honing stage, a second order model was adjusted. The residuals were checked, and a goodness of fit test was performed for each model. For each response, a graphic was obtained (Figure 3, Figure 4 and Figure 5, respectively), in which the coefficients of each regressor are presented in the following way:

- Each horizontal line in the graph corresponds to one of the estimated effects (either the main effect or an interaction) and only the significant effects are represented with a ball.
- The size of the ball is proportional to the absolute value of the coefficient in the fitted model, so the biggest balls represent the effects with highest values.
- The color of the ball corresponds to the sign of the coefficient: red corresponds to positive and blue to negative.

In the following subsections the results for surface roughness, material removal rate and tool wear are presented.

#### 3.1.1. Roughness, Ra

Equations (5)–(7) provide the regression models for Ra in the rough, semi-finish and finish operation respectively.

Ra, rough = 2.64 − 0.00570 Gs + 0.0017 De + 0.00630 Pr + 0.0459 Vt − 0.394 Vl − 0.000848 De^2 − 0.000007 Pr^2 + 0.00653 Vl^2 + 0.000546 Gs·De + 0.000145 Gs·Vt − 0.000283 Gs·Vl − 0.000085 Pr·Vt + 0.000249 Pr·Vl
(5)

Ra, semi-finish = −2.869 + 0.0712 Gs − 0.0500 De + 0.000231 Pr − 0.0053 Vt + 0.1704 Vl − 0.000683 Gs^2 + 0.000866 De^2 − 0.00444 Vl^2 + 0.000019 Gs·Pr + 0.000301 Gs·Vt − 0.000036 De·Pr + 0.000387 De·Vt + 0.000047 Pr·Vl − 0.000896 Vt·Vl
(6)

Ra, finish = 1.165 − 0.07211 Gs − 0.01334 De − 0.000773 Pr − 0.01099 Vt + − 0.00007 Vl + 0.001133 Gs^2 + 0.000627 Gs·De + 0.000036 Gs·Pr + 0.000150 Gs·Vt + 0.000013 Pr·Vt
(7)

**Figure 6:**
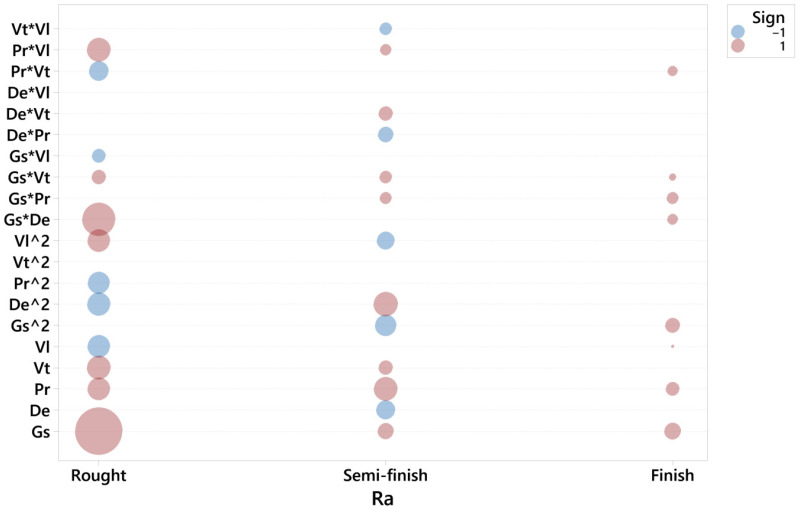
Significant terms for Ra in the rough, semi-finish and finish operations.

Figure 6 depicts the significant terms for the roughness parameter, Ra, in the rough, semi-finish and finish operations.

In the rough honing operation, the main factor influencing roughness is grain size, Gs, followed by Vt, Pr and Vl. The higher the grain size, tangential speed and pressure, the higher roughness is. Conversely, the lower the linear speed, the higher roughness is. The interaction between grain size and density is significant, as has been observed in previous works [10]. The higher the grain size, the higher density should be in order to assure the correct cutting operation. Other significant interactions are Pr·Vl, Pr·Pr and De·De. Lawrence and Ramamoorthy [22] found that rotational speed was the most influential factor on the Rz parameter, followed by oscillatory speed, honing time and pressure. These results are in accordance with the present work, considering that they did not vary grain size nor abrasive density. Gunay and Korkmaz [35] also reported a higher influence of grit size compared to linear speed in honing processes.

In the semi-finish operation, the most significant term becomes De·De, followed by Pr, while the term Gs·Gs is also important. This suggests that, although roughness depends directly on pressure, it is also influenced by grain size and density. The fact that pressure influences roughness is in accordance with the results of Kanthababu et al. [5]. 

In the finish operation, grain size and pressure seem to be the only factors that have an influence on roughness, while density appears in the Gs·De interaction. Gs·Gs, Gs·Pr and Pr·Vt are also influential. Conversely, Bai et al. [13] found that surface roughness depends on tangential speed. In plateau honing processes, Gunay and Korkmaz [35] observed that roughness depended mainly on grain size, linear speed and number of strokes. A grain size of 150, a linear speed of 7 m/min and four strokes are recommended in order to minimize R_a_.

In summary, in rough honing processes it is important to select low grain size and low density to ensure low roughness. In addition to grain size and density, pressure also becomes important in the semi-finish operation. The lower the pressure, the lower roughness is. In the finish operation, the main factor to be considered is grain size, followed by pressure. Thus, in finish honing processes, the density of the abrasive is not so important as in rough and semi-finish processes.

**Figure 7:**
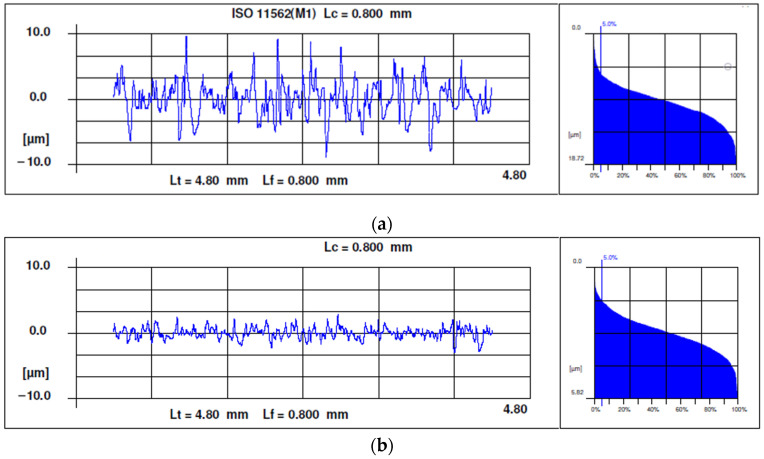

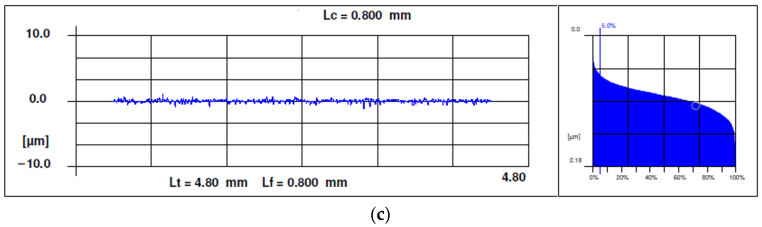
Examples of roughness profiles and Abbott–Firestone curves: (**a**) rough honing with grain size 181, (**b**) semi-finish honing with grain size 76 and (**c**) finish honing with grain size 30.

As an example, Figure 7 shows a roughness profile for Experiment 2 on (a) rough honing, (b) semi-finish honing and (c) finish honing.

All the profiles present sharp peaks and rounded valleys, with an irregular shape that is characteristic of abrasive machining processes. As expected, the higher the grain size, the higher roughness is. The Abbot–Firestone curves have the s-shape that is characteristic of the abrasive machining processes.

#### 3.1.2. Material Removal Rate

Equations (8)–(10) correspond to the regression models for the material removal rate, Qm, in the rough, semi-finish and finish operations, respectively:

Qm, rough = −0.419 − 0.000830 Gs + 0.00801 De + 0.001799 Pr − 0.00387 Vt − 0.00962 Vl − 0.000154 De^2 − 0.000002 Pr^2 + 0.000017 Gs·De + 0.000027 Gs·Vt + 0.000104 De·Vl + 0.000006 Pr·Vt + 0.000016 Pr·Vl
(8)

Qm, semi-finish = −0.3379 + 0.01439 Gs − 0.00465 De + 0.000007 Pr − 0.002380 Vt + 0.000350 Vl − 0.000119 Gs^2 + 0.000063 De^2 + 0.000033 De·Vt + 0.000004 Pr·Vt
(9)

Qm, finish = 0.1621 − 0.001941 Gs − 0.001733 De − 0.000108 Pr − 0.001910 Vt − 0.00958 Vl + 0.000182 Vl^2 + 0.000088 Gs·De + 0.000002 Gs·Pr + 0.000002 Pr·Vt + 0.000003 Pr·Vl + 0.000051 Vt·Vl
(10)

**Figure 8:**
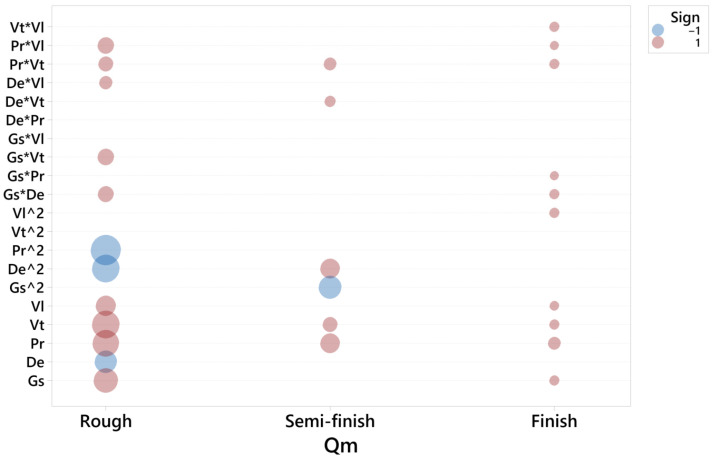
Significant terms for Qm in the rough, semi-finish and finish operations.

Figure 8 corresponds to the models of material removal rate, Qm, in rough, semi-finish and finish processes, respectively.

In the rough honing operation, the most significant term influencing the material removal rate is Pr·Pr, followed by Vt, Pr, De·De and Gs. Thus, pressure seems to be crucial to ensure a sufficient material removal rate in this operation, as has been previously observed [11], although the other parameters are also important in this case. In honing processes with diamond stones of grain size 181 and 151, respectively, Vrac et al. [7] found that cutting speed greatly influenced material removal rate, while pressure was less relevant.

As for the semi-finish operation, the main terms are Gs·Gs, Pr and De·De. This suggests that, as the quantity of material to be removed decreases in subsequent honing operations, the importance of pressure is reduced because the cutting operation becomes easier to perform.

In the finish operation, different factors show a similar impact: Pr, Gs, Vt and Vl. Vl·Vl is also significant, and density appears in the Gs·De interaction.

In summary, all factors influence the material removal rate in rough honing. In semi-finish honing, mainly pressure, grain size and density should be considered, while in finish honing, all factors except density are important.

#### 3.1.3. Tool Wear

Equations (11)–(13) show the regression models for tool wear, Qp, in the rough, semi-finish and finish operations respectively.

Qp, rough = −0.001659 + 0.000001 Gs + 0.000037 De + 0.000003 Pr + 0.000025 Vt + 0.000005 Vl − 0.000000 De^2 − 0.000000 De·Pr − 0.000000 De·Vt
(11)

Qp, semi-finish = −0.000317 + 0.000021 Gs − 0.000015 De + 0.000002 Pr − 0.000019 Vt − 0.000034 Vl − 0.000000 Gs^2 + 0.000000 Gs·De − 0.000000 Gs·Pr + 0.000000 Gs·Vl − 0.000000 De·Pr + 0.000000 De·Vt + 0.000001 Vt·Vl
(12)

Qp, finish = 0.000906 − 0.000095 Gs + 0.000019 De − 0.000001 Pr − 0.000000 Vt + 0.000002 Gs^2 − 0.000001 Gs·De + 0.000000 Gs·Pr + 0.000000 Gs·Vt – 0.000000 De·Vt
(13)

**Figure 9:**
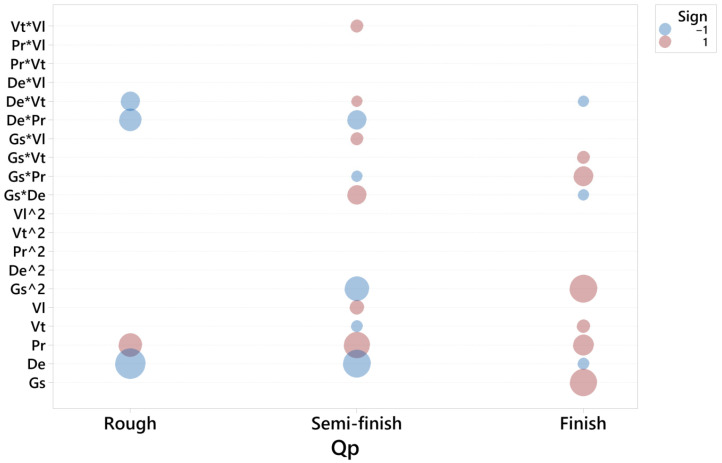
Significant terms for Qp in the rough, semi-finish and finish operations.

Figure 9 depicts the main terms influencing tool wear, Qp, in the rough, semi-finish and finish operations.

The main factor affecting tool wear in rough honing is the density of the abrasive, De, with a negative impact on tool wear. This suggests that a lower density favors the removal of grains from the bond, which restores the stones’ ability to cut but at the cost of increasing tool wear. Other important factors are pressure and the interaction between density and pressure.

In the semi-finish honing operation, density and pressure are still the most important factors. However, a new Gs·Gs starts to influence tool wear with a negative impact: higher grain size leads to lower tool wear.

In the finish operation, grain size is the most important factor influencing tool wear, with the terms Gs and Gs·Gs, followed by pressure and by the interaction between grain size and pressure. 

In summary, the density of the abrasive is a crucial factor in rough honing. However, in semi-finish and finish honing, the grain of the abrasive becomes more important. In all the honing steps, pressure is a factor to be considered.

When using cBN tools, tool wear is characterized by low values. For this reason, tool wear has only a small influence on the performance of the present tests, in which honing time was relatively short.

### 3.2. Multi-Objective Optimization

The main results of the optimization step are presented in Section 3.2.1, Section 3.2.2 and Section 3.3.3 for the rough, semi-finish and finish phases, respectively.

#### 3.2.1. Rough Honing

**Figure 10:**
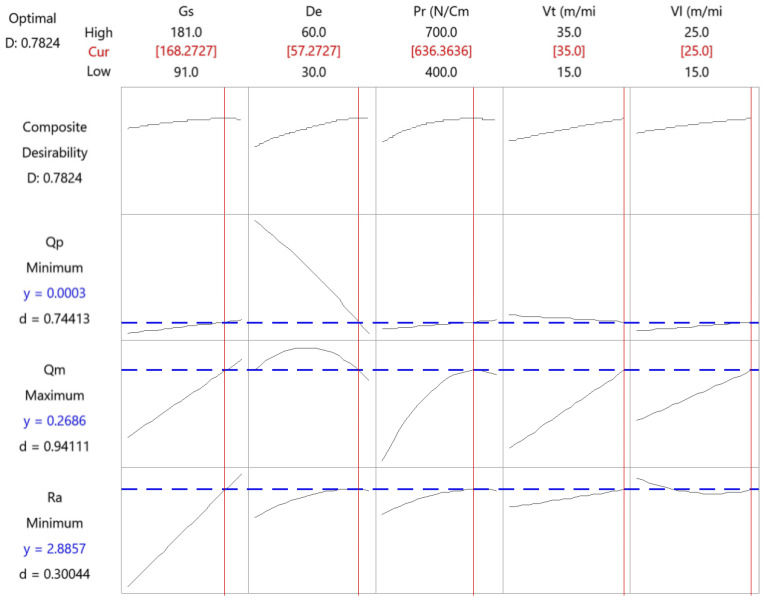
Multi-objective optimization of the rough honing operation.

Figure 10 presents the results of the multi-objective optimization for the rough honing operation.

The combination that minimizes tool wear and roughness while maximizing the material removal rate is presented in Table 3.

This corresponds to medium grain size and high values for the rest of the factors. In rough honing, a high grain size would be recommended in order to provide a high material removal rate, but a low grain size would provide a better surface finish [36]. Thus, medium grain size optimizes both responses.

#### 3.2.2. Semi-Finish Honing

**Figure 11:**
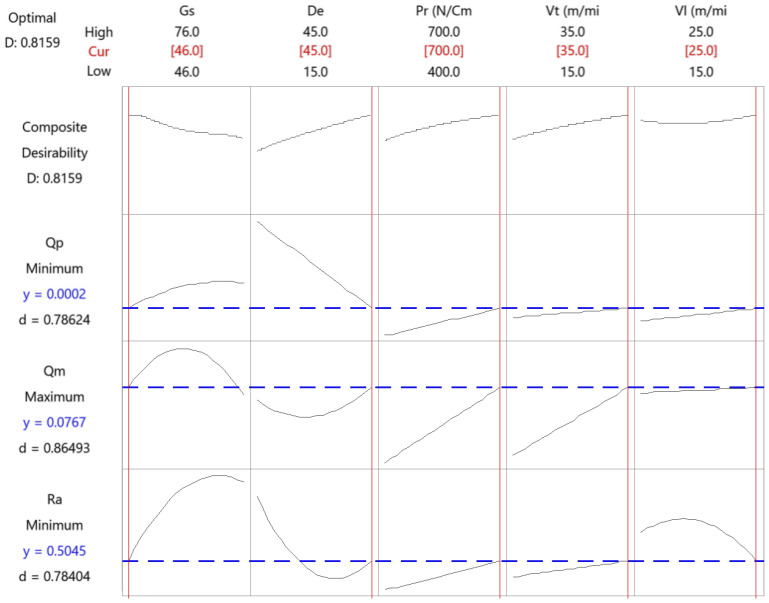
Multi-objective optimization of the semi-finish honing operation.

Figure 11 corresponds to the results of the multi-objective optimization for the semi-finish honing operation.

The combination that minimizes roughness and tool wear and maximizes the material removal rate is shown in Table 4.

This combination includes a low grain size, while the rest of the variables are kept at their high values.

#### 3.2.3. Finish Honing

**Figure 12:**
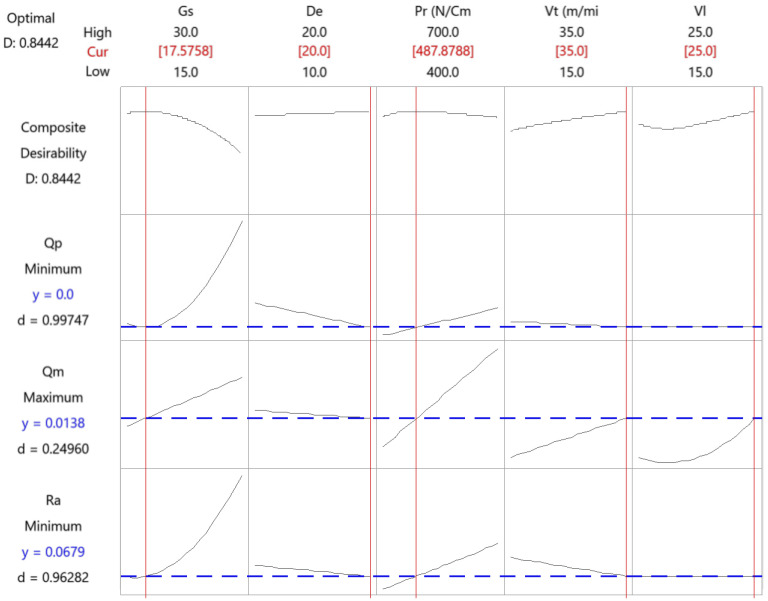
Multi-objective optimization of the finish honing operation.

Figure 12 shows the results for the finish phase.

Table 5 presents the results of the multi-objective optimization in the finish honing operation.

Recommended values for the variables are: low grain size (close to the lower limit of 15), high density, low pressure (close to the lower limit of 400), high tangential speed and high linear speed.

In all the honing phases, high linear and tangential speed values are to be selected.

### 3.3. Sensitivity Analysis

The results of the sensitivity analysis for the rough, semi-finish and finish phase are presented in Section 3.3.1, Section 3.3.2 and Section 3.3.3, respectively.

#### 3.3.1. Rough Honing

**Figure 13:**
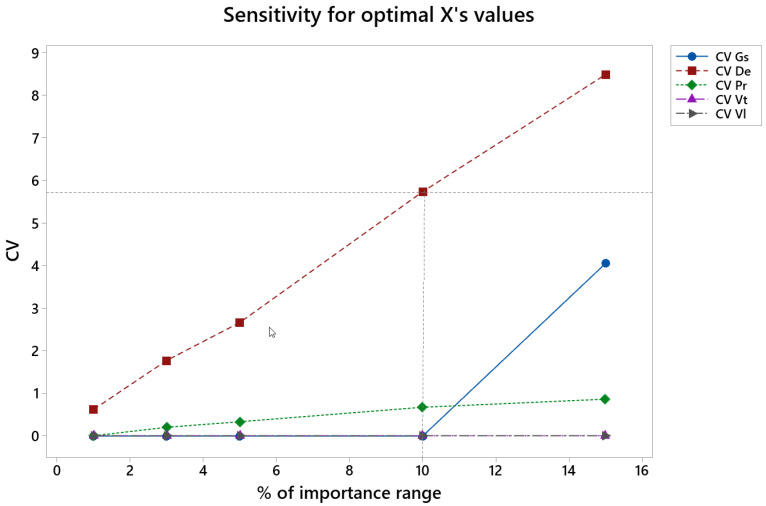
CV of the different factors vs. percentage of importance range in the rough honing operation.

Figure 13 depicts the variation coefficient, CV, vs. the percentage of variation of the importance values in rough honing (% of importance range), from the initial numbers of 0.1 for roughness Ra, 0.6 for material removal rate, Qm, and 0.3 for tool wear (see Table 2).

In the rough honing operation, the coefficient of variation, CV, is lower than 3 for all the factors up to 5% of importance variation and lower than 6 for all the factors up to 10% variation of the importance. CV values are especially low for grain size, pressure, tangential speed and linear speed. CV increases noticeably for De.

#### 3.3.2. Semi-Finish Honing

**Figure 14:**
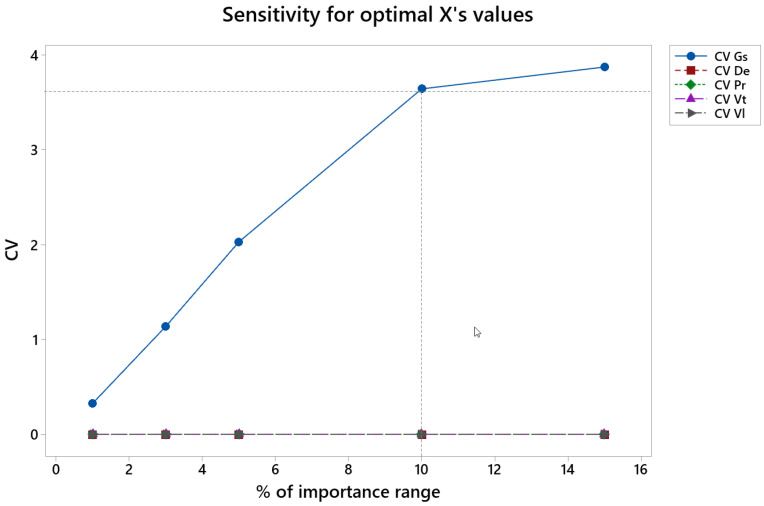
CV of the different factors vs. percentage of importance range in the semi-finish honing operation.

Figure 14 shows the coefficient of variation, CV, vs. percentage of variation of the importance values in semi-finish honing (% of importance range), from the initial numbers of 0.4 for roughness Ra, 0.4 for material removal rate, Qm, and 0.2 for tool wear (see Table 2).

In the semi-finish phase the CV is lower than 3 for all the variables up to 5% of variation in the importance values, and it is lower than 4 up to 10% of variation in the importance values. CV increases greatly for Gs.

#### 3.3.3. Finish Honing

**Figure 15:**
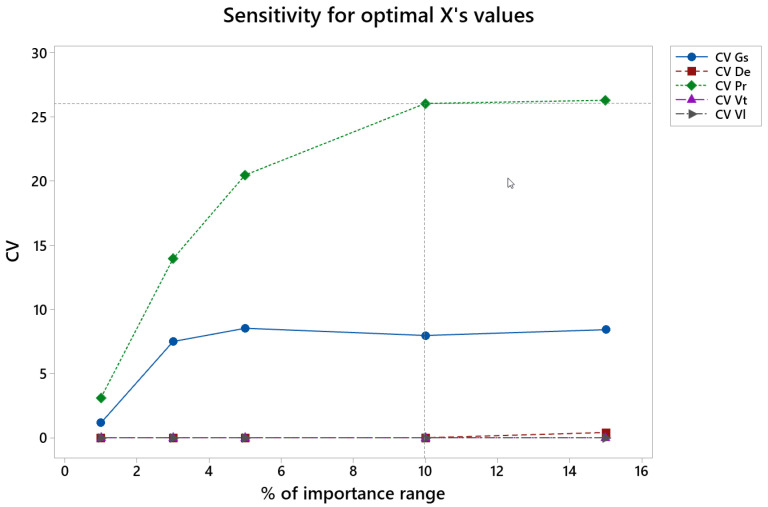
CV of the different factors vs. percentage of importance range in the finish honing operation.

In Figure 15 the CV for all variables vs. the variation of importance values in finish honing (% of importance range) is presented, from the initial numbers of 0.8 for roughness Ra, 0.1 for material removal rate, Qm, and 0.1 for tool wear (see Table 2).

In the finish phase the CV is lower than 10 for all the variables up to 5% of variation in the importance values, except for pressure, for which CV increases noticeably up to a variation of 10% in the importance value.

## 4. Conclusions

In the present paper, regression models are presented for roughness, material removal rate and tool wear in the rough, semi-finish and finish operations. A sensitivity study is presented for the multi-objective optimization process. The main conclusions of the work are as follows:

- Grain size is the most influential factor on roughness, while pressure influences the material removal rate in all the honing steps.
- In order to minimize roughness and tool wear, and to maximize the material removal rate, medium or high values for the different variables are recommended in the rough phase. In the semi-finish phase, low grain size is recommended, while the rest of the variables should be held at high values. In the finish phase, low grain size and pressure are recommended, with high values for the rest of the variables.
- The sensitivity analysis showed that, when performing a multi-objective optimization in the rough and in the semi-finish phases, variations of the importance values for each response that are lower than 5% do not significantly increase the variation coefficient of the different variables. This means one can reasonably decide on the importance for each response in the rough and semi-finish phases, being confident that mild changes in these importance values will not have a large effect. Conversely, in the finish phase, small changes in the importance values increase the variation coefficient of pressure. Thus, it is recommended to select accurately the importance values of the different responses in the finish phase.

## Figures and Tables

**Table 1:** Low and high levels for the different variables employed in the rough, semi-finish and finish experiments.

	Rough	Semi-Finish	Finish
Grain size, Gs (ISO 6106 [33])	91–181	46–76	15–30
Density of abrasive, De (ISO 6104 [34])	30–60	15–45	10–20
Pressure, Pr (N/cm^2^)	400–700	400–700	400–700
Tangential speed, Vt (m/min)	30–50	30–50	30–50
Linear speed, Vl (m/min)	20–40	20–40	20–40

**Table 2:** Importance values used for each response and honing phase in the optimization.

Response	Rough	Semi-Finish	Finish
Average roughness, Ra (µm)	0.1	0.4	0.8
Material removal rate, Qm (cm/min)	0.6	0.4	0.1
Tool wear, Qp (cm^3^/min)	0.3	0.2	0.1

**Table 3:** Results of the multi-objective optimization in the rough honing operation.

Parameter	Gs	De	Pr	Vt	Vl
Value	168	57	636	35	25

**Table 4:** Results of the multi-objective optimization in the semi-finish honing operation.

Parameter	Gs	De	Pr	Vt	Vl
Value	46	45	700	35	25

**Table 5:** Results of the multi-objective optimization in the finish honing operation.

Parameter	Gs	De	Pr	Vt	Vl
Value	17	20	488	35	25

## Data Availability

The data presented in this study are available in Appendix A.

## Appendix A

**Table A1:** Experimentation results in the rough phase.

Run	Gs	De	Pr (N/cm^2^)	Vt (m/min)	Vl (m/min)	Ra (µm)	Qm (cm/min)	Qp (cm^3^/min)
1	91	30	400	15	25	0.91	0.0822	0.000249
2	91	30	400	15	25	0.78	0.0602	0.000167
3	91	30	400	35	15	1.90	0.1151	0.000167
4	91	30	400	35	15	1.51	0.1260	0.000488
5	91	30	700	15	15	1.48	0.1314	0.000381
6	91	30	700	15	15	1.37	0.1041	0.000716
7	91	30	700	35	25	1.67	0.2374	0.001010
8	91	30	700	35	25	1.59	0.2305	0.000652
9	91	45	550	25	20	1.22	0.1540	0.000367
10	91	45	550	25	20	1.20	0.1589	0.000346
11	91	60	400	15	15	0.61	0.0219	0.000225
12	91	60	400	15	15	0.62	0.0273	0.000149
13	91	60	400	35	25	0.59	0.0602	0.000029
14	91	60	400	35	25	0.52	0.0547	0.000087
15	91	60	700	15	25	0.70	0.0929	0.000214
16	91	60	700	15	25	0.81	0.0929	0.000062
17	91	60	700	35	15	0.82	0.0715	0.000116
18	91	60	700	35	15	0.78	0.0767	0.000029
19	126	30	550	25	20	1.76	0.1375	0.000356
20	126	30	550	25	20	1.88	0.1811	0.000990
21	126	45	400	25	20	1.54	0.1255	0.000361
22	126	45	400	25	20	1.33	0.1036	0.000195
23	126	45	550	15	20	1.61	0.1478	0.000722
24	126	45	550	15	20	1.80	0.1476	0.000086
25	126	45	550	25	15	2.42	0.1642	0.000264
26	126	45	550	25	15	2.00	0.1534	0.000562
27	126	45	550	25	20	1.85	0.2402	0.000300
28	126	45	550	25	20	1.76	0.1910	0.000452
29	126	45	550	25	20	1.81	0.1965	0.000511
30	126	45	550	25	20	1.93	0.2075	0.000274
31	126	45	550	25	20	1.99	0.2184	0.000447
32	126	45	550	25	20	1.90	0.2238	0.000473
33	126	45	550	25	20	1.83	0.1865	0.000561
34	126	45	550	25	20	1.91	0.1809	0.000461
35	126	45	550	25	20	1.72	0.2303	0.000318
36	126	45	550	25	20	1.72	0.1753	0.000342
37	126	45	550	25	25	1.79	0.2293	0.000581
38	126	45	550	25	25	1.76	0.2238	0.000137
39	126	45	550	35	20	2.21	0.1978	0.000333
40	126	45	550	35	20	1.73	0.2032	0.000652
41	126	45	700	25	20	1.70	0.1531	0.000423
42	126	45	700	25	20	2.08	0.2019	0.000513
43	126	60	550	25	20	1.55	0.1425	0.000532
44	126	60	550	25	20	1.36	0.1527	0.000497
45	181	30	400	15	15	2.11	0.0872	0.000219
46	181	30	400	15	15	1.84	0.0818	0.000232
47	181	30	400	35	25	2.10	0.1855	0.000562
48	181	30	400	35	25	1.77	0.1527	0.000599
49	181	30	700	15	25	2.19	0.1528	0.000807
50	181	30	700	15	25	2.55	0.1582	0.000689
51	181	30	700	35	15	2.14	0.1965	0.000808
52	181	30	700	35	15	2.86	0.2841	0.000915
53	181	45	550	25	20	2.57	0.2349	0.000315
54	181	45	550	25	20	2.80	0.2512	0.000416
55	181	60	400	15	25	1.96	0.0873	0.000189
56	181	60	400	15	25	1.78	0.0872	0.000231
57	181	60	400	35	15	3.90	0.1255	0.000068
58	181	60	400	35	15	3.47	0.1146	0.000126
59	181	60	700	15	15	3.23	0.0875	0.000184
60	181	60	700	15	15	2.79	0.0765	0.000184
61	181	60	700	35	25	3.16	0.2571	0.000384
62	181	60	700	35	25	3.21	0.2514	0.000076

**Table A2:** Experimentation results in the semi-finish phase.

Run	Gs	De	Pr (N/cm^2^)	Vt (m/min)	Vl (m/min)	Ra (µm)	Qm (cm/min)	Qp (cm^3^/min)
1	46	15	400	15	25	0.47	0.0183	3.82 × 10^−4^
2	46	15	400	15	25	0.49	0.0258	2.68 × 10^−4^
3	46	15	400	35	15	0.51	0.0183	2.54 × 10^−4^
4	46	15	400	35	15	0.47	0.0183	1.40 × 10^−4^
5	46	15	700	15	15	0.81	0.0403	8.98 × 10^−4^
6	46	15	700	15	15	0.79	0.0477	7.48 × 10^−4^
7	46	15	700	35	25	0.84	0.0587	7.42 × 10^−4^
8	46	15	700	35	25	0.82	0.0622	7.28 × 10^−4^
9	46	30	550	25	20	0.49	0.0331	2.57 × 10^−4^
10	46	30	550	25	20	0.46	0.0405	1.85 × 10^−4^
11	46	45	400	15	15	0.27	0.0110	0.00
12	46	45	400	15	15	0.30	0.0073	6.07 × 10^−5^
13	46	45	400	35	25	0.39	0.0293	8.62 × 10^−18^
14	46	45	400	35	25	0.38	0.0220	4.85 × 10^−5^
15	46	45	700	15	25	0.36	0.0221	1.46 × 10^−4^
16	46	45	700	15	25	0.58	0.0481	1.74 × 10^−4^
17	46	45	700	35	15	0.57	0.0733	1.04 × 10^−4^
18	46	45	700	35	15	0.58	0.0733	9.71 × 10^−5^
19	64	15	550	25	20	0.86	0.0402	5.63 × 10^−4^
20	64	15	550	25	20	1.02	0.0699	4.52 × 10^−4^
21	64	30	400	25	20	0.37	0.0147	2.65 × 10^−4^
22	64	30	400	25	20	0.31	0.0220	1.68 × 10^−4^
23	64	30	550	15	20	0.71	0.0475	4.30 × 10^−4^
24	64	30	550	15	20	0.65	0.0440	3.04 × 10^−4^
25	64	30	550	25	15	0.57	0.0330	3.09 × 10^−4^
26	64	30	550	25	15	0.54	0.0293	4.01 × 10^−4^
27	64	30	550	25	20	0.96	0.0661	2.95 × 10^−4^
28	64	30	550	25	20	0.77	0.0366	3.09 × 10^−4^
29	64	30	550	25	20	0.91	0.0403	3.82 × 10^−4^
30	64	30	550	25	20	0.74	0.0549	2.61 × 10^−4^
31	64	30	550	25	20	0.66	0.0476	3.09 × 10^−4^
32	64	30	550	25	20	0.72	0.0439	3.30 × 10^−4^
33	64	30	550	25	20	0.56	0.0366	3.51 × 10^−4^
34	64	30	550	25	20	0.66	0.0547	3.63 × 10^−4^
35	64	30	550	25	20	0.76	0.0583	3.82 × 10^−4^
36	64	30	550	25	20	0.60	0.0404	2.69 × 10^−4^
37	64	30	550	25	25	0.59	0.0440	3.95 × 10^−4^
38	64	30	550	25	25	0.64	0.0512	4.04 × 10^−4^
39	64	30	550	35	20	0.65	0.0588	3.57 × 10^−4^
40	64	30	550	35	20	0.70	0.0550	2.80 × 10^−4^
41	64	30	700	25	20	1.09	0.0661	5.39 × 10^−4^
42	64	30	700	25	20	1.02	0.0584	5.86 × 10^−4^
43	64	45	550	25	20	0.89	0.0658	1.71 × 10^−4^
44	64	45	550	25	20	0.79	0.0806	2.03 × 10^−4^
45	76	15	400	15	15	0.57	0.0146	1.27 × 10^−4^
46	76	15	400	15	15	0.62	0.0146	2.75 × 10^−4^
47	76	15	400	35	25	0.47	0.0111	2.52 × 10^−4^
48	76	15	400	35	25	0.47	0.0257	2.29 × 10^−4^
49	76	15	700	15	25	1.06	0.0440	6.70 × 10^−4^
50	76	15	700	15	25	1.07	0.0475	5.65 × 10^−4^
51	76	15	700	35	15	1.27	0.0698	4.42 × 10^−4^
52	76	15	700	35	15	1.37	0.0734	4.19 × 10^−4^
53	76	30	550	25	20	0.47	0.0146	2.16 × 10^−4^
54	76	30	550	25	20	0.70	0.0109	2.72 × 10^−4^
55	76	45	400	15	25	0.27	0.0037	1.91 × 10^−4^
56	76	45	400	15	25	0.27	0.0183	2.83 × 10^−4^
57	76	45	400	35	15	0.75	0.0293	0.00
58	76	45	400	35	15	0.69	0.0219	1.21 × 10^−4^
59	76	45	700	15	15	0.38	0.0110	2.31 × 10^−4^
60	76	45	700	15	15	0.50	0.0147	1.09 × 10^−4^
61	76	45	700	35	25	0.65	0.0440	3.56 × 10^−4^
62	76	45	700	35	25	1.07	0.0881	4.36 × 10^−4^

**Table A3:** Experimentation results in the finish phase.

Run	Gs	De	Pr (N/cm^2^)	Vt (m/min)	Vl (m/min)	Ra (µm)	Qm (cm/min)	Qp (cm^3^/min)
1	15	10	400	15	25	0.24	0.0037	1.76 × 10^−5^
2	15	10	400	15	25	0.09	0.0037	1.04 × 10^−5^
3	15	10	400	35	15	0.06	0.0037	2.27 × 10^−5^
4	15	10	400	35	15	0.09	0.0037	0.00
5	15	10	700	15	15	0.14	0.0110	3.09 × 10^−5^
6	15	10	700	15	15	0.16	0.0110	5.88 × 10^−5^
7	15	10	700	35	25	0.19	0.0313	9.41 × 10^−5^
8	15	10	700	35	25	0.12	0.0313	7.71 × 10^−5^
9	15	15	550	25	20	0.12	0.0037	4.15 × 10^−5^
10	15	15	550	25	20	0.09	0.0055	8.30 × 10^−5^
11	15	20	400	15	15	0.11	0.0037	0.00
12	15	20	400	15	15	0.09	0.0037	1.15 × 10^−5^
13	15	20	400	35	25	0.06	0.0074	0.00
14	15	20	400	35	25	0.05	0.0073	3.46 × 10^−5^
15	15	20	700	15	25	0.15	0.0092	6.95 × 10^−5^
16	15	20	700	15	25	0.09	0.0073	3.37 × 10^−5^
17	15	20	700	35	15	0.09	0.0093	2.31 × 10^−5^
18	15	20	700	35	15	0.14	0.0166	9.88 × 10^−6^
19	20	10	550	25	20	0.11	0.0037	1.13 × 10^−5^
20	20	10	550	25	20	0.09	0.0037	1.13 × 10^−5^
21	20	15	400	25	20	0.11	0.0037	2.76 × 10^−5^
22	20	15	400	25	20	0.10	0.0037	6.22 × 10^−5^
23	20	15	550	15	20	0.11	0.0074	3.61 × 10^−5^
24	20	15	550	15	20	0.12	0.0092	4.66 × 10^−5^
25	20	15	550	25	15	0.15	0.0092	4.15 × 10^−5^
26	20	15	550	25	15	0.12	0.0092	4.15 × 10^−5^
27	20	15	550	25	20	0.12	0.0037	5.92 × 10^−5^
28	20	15	550	25	20	0.15	0.0037	7.10 × 10^−5^
29	20	15	550	25	20	0.12	0.0037	0.00
30	20	15	550	25	20	0.11	0.0055	6.00 × 10^−5^
31	20	15	550	25	20	0.12	0.0055	8.08 × 10^−5^
32	20	15	550	25	20	0.14	0.0074	1.10 × 10^−4^
33	20	15	550	25	20	0.11	0.0055	1.08 × 10^−4^
34	20	15	550	25	20	0.12	0.0055	3.63 × 10^−5^
35	20	15	550	25	20	0.12	0.0091	2.66 × 10^−5^
36	20	15	550	25	20	0.12	0.0091	5.32 × 10^−5^
37	20	15	550	25	25	0.11	0.0129	2.30 × 10^−5^
38	20	15	550	25	25	0.12	0.0148	5.54 × 10^−5^
39	20	15	550	35	20	0.10	0.0092	7.92 × 10^−5^
40	20	15	550	35	20	0.11	0.0092	1.11 × 10^−4^
41	20	15	700	25	20	0.17	0.0127	2.27 × 10^−5^
42	20	15	700	25	20	0.16	0.0111	0.00
43	20	20	550	25	20	0.11	0.0055	5.77 × 10^−5^
44	20	20	550	25	20	0.08	0.0055	5.77 × 10^−5^
45	30	10	400	15	15	0.18	0.0018	6.03 × 10^−5^
46	30	10	400	15	15	0.16	0.0037	1.61 × 10^−4^
47	30	10	400	35	25	0.16	0.0037	2.84 × 10^−4^
48	30	10	400	35	25	0.15	0.0037	2.64 × 10^−4^
49	30	10	700	15	25	0.33	0.0165	4.11 × 10^−4^
50	30	10	700	15	25	0.31	0.0182	4.32 × 10^−4^
51	30	10	700	35	15	0.34	0.0350	6.60 × 10^−4^
52	30	10	700	35	15	0.50	0.0056	6.60 × 10^−4^
53	30	15	550	25	20	0.33	0.0203	4.00 × 10^−4^
54	30	15	550	25	20	0.32	0.0184	4.45 × 10^−4^
55	30	20	400	15	25	0.26	0.0091	1.61 × 10^−4^
56	30	20	400	15	25	0.25	0.0093	0.00
57	30	20	400	35	15	0.19	0.0074	1.28 × 10^−4^
58	30	20	400	35	15	0.21	0.0073	1.28 × 10^−4^
59	30	20	700	15	15	0.38	0.0184	3.21 × 10^−4^
60	30	20	700	15	15	0.41	0.0165	4.55 × 10^−4^
61	30	20	700	35	25	0.42	0.0424	3.33 × 10^−4^
62	30	20	700	35	25	0.43	0.0497	6.13 × 10^−4^

## References

[1] Lawrence K.D., Ramamoorthy B. (2014). Structure function-based fractal characterisation of cylinder bore surfaces using stylus profile data. Int. J. Precis. Technol..

[2] Grabon W., Pawlus P., Wos S., Koszela W., Wieczorowski M. (2017). Effects of honed cylinder liner surface texture on tribological properties of piston ring-liner assembly in short time tests. Tribol. Int..

[3] Kim E.S., Kim S.M., Lee Y.Z. (2018). The effect of plateau honing on the friction and wear of cylinder liners. Wear.

[4] Troglio A.J. Performance evaluation of multi-stone honing tool by experimental design methods. Proceedings of the International Honing Conference.

[5] Kanthababu M., Shunmugam M.S., Singaperumal M. (2009). Identification of significant parameters and appropriate levels in honing of cylinder liners. Int. J. Mach. Mach. Mater..

[6] Woś P., Michalski J. (2011). Effect of Initial Cylinder Liner Honing Surface Roughness on Aircraft Piston Engine Performances. Tribol. Lett..

[7] Vrac D.S., Sidjanin L.P., Kovac P.P., Balos S.S. (2013). The influence of honing process parameters on surface quality, productivity, cutting angle and coefficients of friction. Ind. Lubr. Tribol..

[8] Vrac D.S., Sidjanin L.P., Balos S.S. (2013). Mechanical finishing honing: Cutting regimes and surface texture. Ind. Lubr. Tribol..

[9] Vrabel’ M., Maňková I., Durakbasa N.M. (2017). Effect of Honing Parameters on Generated Surface Quality of Cylinder Liner within Automotive Engine Production. Solid State Phenom..

[10] Buj-Corral I., Vivancos-Calvet J., Coba-Salcedo M. (2014). Modelling of surface finish and material removal rate in rough honing. Precis. Eng..

[11] Szabo O. (2014). Examination of Material Removal Process in Honing: Ebscohost. Acta Tech. Corviniensis-Bull. Eng..

[12] Bai Y.J., Zhang L.H., Ren C.G. (2007). Experimental investigation on honing of small holes. Key Eng. Mater..

[13] Buj-Corral I., Álvarez-Flórez J., Domínguez-Fernández A. (2019). Effect of grain size and density of abrasive on surface roughness, material removal rate and acoustic emission signal in rough honing processes. Metals.

[14] Arantes L.J., Fernandes K.A., Schramm C.R., Leal J.E.S., Piratelli-Filho A., Franco S.D., Arencibia R.V. (2017). The roughness characterization in cylinders obtained by conventional and flexible honing processes. Int. J. Adv. Manuf. Technol..

[15] Cabanettes F., Dimkovski Z., Rosén B.-G. (2015). Roughness variations in cylinder liners induced by honing tools’ wear. Precis. Eng..

[16] Derringer G., Suich R. (2018). Simultaneous Optimization of Several Response Variables. J. Qual. Technol..

[17] Chabbi A., Yallese M.A., Meddour I., Nouioua M., Mabrouki T., Girardin F. (2017). Predictive modeling and multi-response optimization of technological parameters in turning of Polyoxymethylene polymer (POM C) using RSM and desirability function. Measurement.

[18] Aggarwal A., Singh H., Kumar P., Singh M. (2008). Optimization of multiple quality characteristics for CNC turning under cryogenic cutting environment using desirability function. J. Mater. Process. Technol..

[19] Selaimia A.-A., Yallese M.A., Bensouilah H., Meddour I.K., Khattabi R., Mabrouki T. (2017). Modeling and optimization in dry face milling of X2CrNi18-9 austenitic stainless steel using RMS and desirability approach. Measurement.

[20] Mia M. (2017). Multi-response optimization of end milling parameters under through-tool cryogenic cooling condition. Measurement.

[21] Mukherjee I., Ray P.K. (2008). Optimal process design of two-stage multiple responses grinding processes using desirability functions and metaheuristic technique. Appl. Soft Comput..

[22] Lawrence K.D., Ramamoorthy B. (2016). Multi-surface topography targeted plateau honing for the processing of cylinder liner surfaces of automotive engines. Appl. Surf. Sci..

[23] Nguyen T.T., Vu T.C., Duong Q.D. (2020). Multi-responses optimization of finishing honing process for surface quality and production rate. J. Brazilian Soc. Mech. Sci. Eng..

[24] Castillo E., Mínguez R., Castillo C. (2008). Sensitivity analysis in optimization and reliability problems. Reliab. Eng. Syst. Saf..

[25] Aksezer C.S. (2008). On the sensitivity of desirability functions for multiresponse optimization. J. Ind. Manag. Optim..

[26] Malenović A., Dotsikas Y., Mašković M., Jančić-Stojanović B., Ivanović D., Medenica M. (2011). Desirability-based optimization and its sensitivity analysis for the perindopril and its impurities analysis in a microemulsion LC system. Microchem. J..

[27] Rocha L.C.S., de Paiva A.P., Rotela Junior P., Balestrassi P.P., da Silva Campos P.H. (2017). Robust multiple criteria decision making applied to optimization of AISI H13 hardened steel turning with PCBN wiper tool. Int. J. Adv. Manuf. Technol..

[28] Mudhukrishnan M., Hariharan P., Palanikumar K., Latha B. (2019). Optimization and sensitivity analysis of drilling parameters for sustainable machining of carbon fiber–reinforced polypropylene composites. J. Thermoplast. Compos. Mater..

[29] Shi C., Wu Z., Lv K., Wu L. (2015). A review on mixture design methods for self-compacting concrete. Constr. Build. Mater..

[30] Buruk Sahin Y., Aktar Demirtaş E., Burnak N. (2016). Mixture design: A review of recent applications in the food industry. Pamukkale Univ. J. Eng. Sci..

[31] Misra J.P., Jain P.K., Dwivedi D.K., Mehta N.K. (2013). Mixture D-optimal design of electrolyte composition in ECH of bevel gears. Adv. Mater. Res..

[32] (2021). Geometrical product specifications (GPS)—Surface texture: Profile—Part 2: Terms, Definitions and Surface Texture Parameters.

[33] (2013). Abrasive Products—Checking the Grain Size of Superabrasives.

[34] (2005). Superabrasive Products—Rotating Grinding Tools with Diamond or Cubic Boron Nitride—General Survey, Designation and Multilingual Nomenclature.

[35] Günay M., Korkmaz M.E. (2017). Optimization of honing parameters for renewal of cylinder liners. Gazi Univ. J. Sci..

[36] Buj-Corral I., Álvarez-Flórez J., Domínguez-Fernández A. (2018). Acoustic emission analysis for the detection of appropriate cutting operations in honing processes. Mech. Syst. Signal Process..

